# Miscellaneous

**Published:** 1887-09

**Authors:** 


### MISCELLANEOUS.

Sleep a Preventive of Headache.—A writer in the *Scientific American* says: "Sleep, if taken at the right moment, will prevent an attack of nervous headache. If the subjects of such headaches will watch the symptoms of its coming, they can notice that it begins with a feeling of weariness or heaviness. This is the time a sleep of an hour, or even two, as nature guides, will eventually prevent the headache. If not taken just then, it will be too late; for, after the attack is fairly under way, it is impossible to get sleep till far into the night. It is so common in these days for doctors to forbid having their patients waked to take medicine if they are asleep when the hour comes around, that the people have learned the lesson pretty well, and they generally know that sleep is better for the sick than medicine. But it is not so well known that sleep is a wonderful preventive of disease—better than tonic regulators and stimulants."

Advice on Everyday Housekeeping Topics Given by an Old Housekeeper.—Dr. W. H. Morse, the author of the text book on "New Therapeutical Agents," writes as follows concerning the value of hypophosphites: "There is no question in my mind as to the efficacy of the hypophosphites in curing and preventing pulmonary consumption, as their administration supplies the system with the oxydizable phosphorus, the deficiency of which is the proximate cause of the disease. There may be failures when some of the inferior kinds of the drug are used, but Winchester's hypophosphites of lime and soda, the only perfect preparation, never fails. It has been made since 1858, and in common with other medical men I am confident that its power in the system is unequalled by any other agent. The most I can say is that its use will abolish the greatest scourge of humanity if persisted in, and that it is most excellent for general debility and for weak, puny children. For more particular facts ask any physician or obtain 'Winchester on Pulmonary Consumption,' published at 162 William street, New York."

Decayed Oranges.—Thirty thousand boxes of decayed oranges and lemons, and thirty-five hundred crates of tomatoes, which arrived at New York the other day from the Mediterranean and the south, have been carried out to sea on the Health Department scow and dumped. A worse looking and more offensive smelling lot of stuff was never landed. The loss on the cargo was about $45,000. It is stated that the health authorities will redouble their efforts to have Mediterranean fruit vessels closely inspected. They have had their eyes opened and realize that the ships should be looked after as closely as slaughter houses. The better class of fruit dealers will be glad if the system of inspection is improved.

Five Months Without Food or Drink.—Mary Baker, the fasting girl of Monon, White County, Ind., lay in a trance one hundred and sixteen days without taking either food or drink. Her fast is regarded by medical men as the most wonderful on record, as she is said to have neither eaten nor drank during the long period named. The change in her condition took place when death was expected.

Don't forget the use of Lactated Food, prepared by Wells and Richardson Co., of Burlington, Vt.

As a remedy for obstinate constipation, we hear the best accounts of the *Acid Mannate* manufactured by the *Rio Chemical Co., St. Louis,* and advertised in our Journal. Dr. E. A. Scott of Columbus, Kan., writes of it as follows:

"I have a patient, a man who has been constipated four years, has called upon all the physicians in the place, neither have benefited him, never having an action upon the bowels oftener than six to eight days. He is now taking the Acid Mannate, small doses daily, keeping his bowels free. I have a lady patient who is suffering with a uterine trouble, and has periodical nervous sick headache (I think solely dependent upon the uterine trouble), she is also constipated. I have her upon the Acid Mannate; she, as well as myself, is pleased with its effect; her headaches are not so frequent or severe."

### THE BOYS IN BLUE.

Clara was fond of the boys in blue,
For George, *her George*, was a guardsman, too.

En route for the mimic seat of war,
He had called to say his *au revoir*.

And so you are bound for the camp, to-day,
What do you there such a while away?

We drill-parade, mount guard and mess,
And lounge about in our undress.

Oh, la! what dreadful funny men,
And do you let folks see you then?

When they sound the taps, my dear, you know
It means lights out and visitors *go*.

But what if they're naughty, oh, ever so bad
And only make fun of the orders had?

Why there's the guard awaiting alarms,
To seize and hold them under arms.

How awful nice if it only were *me*,
I'd be as naughty as I could be.

De Travers.

